# Ferroptosis: A Potential Target in Cardiovascular Disease

**DOI:** 10.3389/fcell.2021.813668

**Published:** 2022-01-20

**Authors:** Yanlong Leng, Xing Luo, Jiaying Yu, Haibo Jia, Bo Yu

**Affiliations:** ^1^ Department of Cardiology, The Second Affiliated Hospital of Harbin Medical University, Harbin, China; ^2^ Key Laboratory of Myocardial Ischemia, Harbin Medical University, Harbin, China; ^3^ Department of Obstetrics and Gynecology, The Second Affiliated Hospital of Harbin Medical University, Harbin, China

**Keywords:** ferroptosis, cardiovascular disease, iron homeostasis, antioxidant metabolism, lipid metabolism, ischemia reperfusion, heart failure, coronary atherosclerotic heart disease

## Abstract

Ferroptosis is a new form of regulatory cell death characterized by iron-dependent and intracellular lipid peroxidation. Ferroptosis can be divided into two stages. The first stage is iron overload in the cell, which generates a large amount of reactive oxygen species through the Fenton reaction, and the second stage results from an imbalance of the intracellular antioxidant system. Excessive phospholipid hydroperoxides cannot be removed by reduction reactions, as this could destroy the cell membrane structure and interfere with mitochondrial function, eventually leading to ferroptosis of the cell. Cardiovascular diseases have gradually become the leading cause of death in modern society. The relationship between ferroptosis and the occurrence and progression of cardiovascular disease has become a research hotspot in recent years. In this review, we summarize the mechanism of ferroptosis and its specific role in cardiovascular disease.

## Introduction

Cell death is an important cornerstone for maintaining the balance of development and homeostasis of the body. There are two main types of cell death: regulatory cell death (RCD) and non-regulatory cell death (Lockshin and Zakeri, 2001). Nonregulatory cell death refers mainly to the severe damage of cells under the stimulation of various pathogenic factors, leading to passive cell death. However, according to the different signaling pathways involved in the process, regulatory cell death can be divided into apoptosis, necroptosis, pyroptosis, autophagy, and ferroptosis (Del Re et al., 2019). Among them, ferroptosis is a new type of regulatory cell death discovered only recently. In fact, in 2003, Dolma et al. (Dolma et al., 2003) identified a new type of compound that caused tumor cell death and named it erastin. However, due to limitations in research on regulatory cell death at that time, the authors failed to identify the special regulatory cell death mode induced by erastin. Until 2012, when Dixon et al. defined erastin-induced cell death as iron-dependent regulatory cell death induced by lipid peroxidation and named it ferroptosis (Dixon et al., 2012). The morphological characteristics of ferroptosis cells are as follows: mitochondrial shrinkage, decreased mitochondrial cristae, and decreased membrane density, which are different from the characteristic apoptotic body formation of apoptotic cells.

Cardiovascular disease is one of the most threatening diseases to human health today. Cardiovascular diseases mainly include coronary atherosclerotic heart disease, myocardial ischemia/reperfusion injury (IRI), and heart failure (HF). Severe coronary artery stenosis can lead to IRI after recanalization, HF after myocardial injury, and even ischemic cardiomyopathy (Ezekowitz et al., 2009). Therefore, a clear understanding of the pathophysiological basis behind the onset of cardiovascular diseases is of great significance for the prevention, early diagnosis, and precise treatment of cardiovascular diseases. At present, a variety of regulatory cell deaths are thought to regulate the death of related cells in cardiovascular disease (Choi et al., 2019; Dong et al., 2019; Zhaolin et al., 2019). Ferroptosis, as an emerging mode of regulatory cell death, has become a new focus of research in the field of cardiovascular disease. Understanding the mechanism by which ferroptosis occurs or is inhibited in cardiovascular disease is a prerequisite to reducing morbidity and mortality in the future. In this review, we enumerate the latest developments in different aspects of the mechanism of ferroptosis and then will specifically review the unique pathological characteristics of ferroptosis in different cardiovascular diseases, which will be a reference for the prevention and treatment strategies.

## Regulation of Iron Homeostasis

Iron is an essential trace element for the human body; it participates in the formation of a variety of proteins, including hemoglobin, myoglobin, and ferritin, and various redox reactions in cells. It is not only involved in the above-mentioned biological processes but is also the most important initiation and promotion factor for ferroptosis. Under physiological conditions, the complex and precise mechanism of iron homeostasis regulation ensures that iron concentration in the cell remains stable and prevents intracellular iron overload from triggering the Fenton reaction, which in turn leads to ferroptosis (Ganz, 2013). The regulation of iron homeostasis mainly includes mechanisms involving import, export, storage, and utilization of iron.

### Iron Export

**FIGURE 1:**
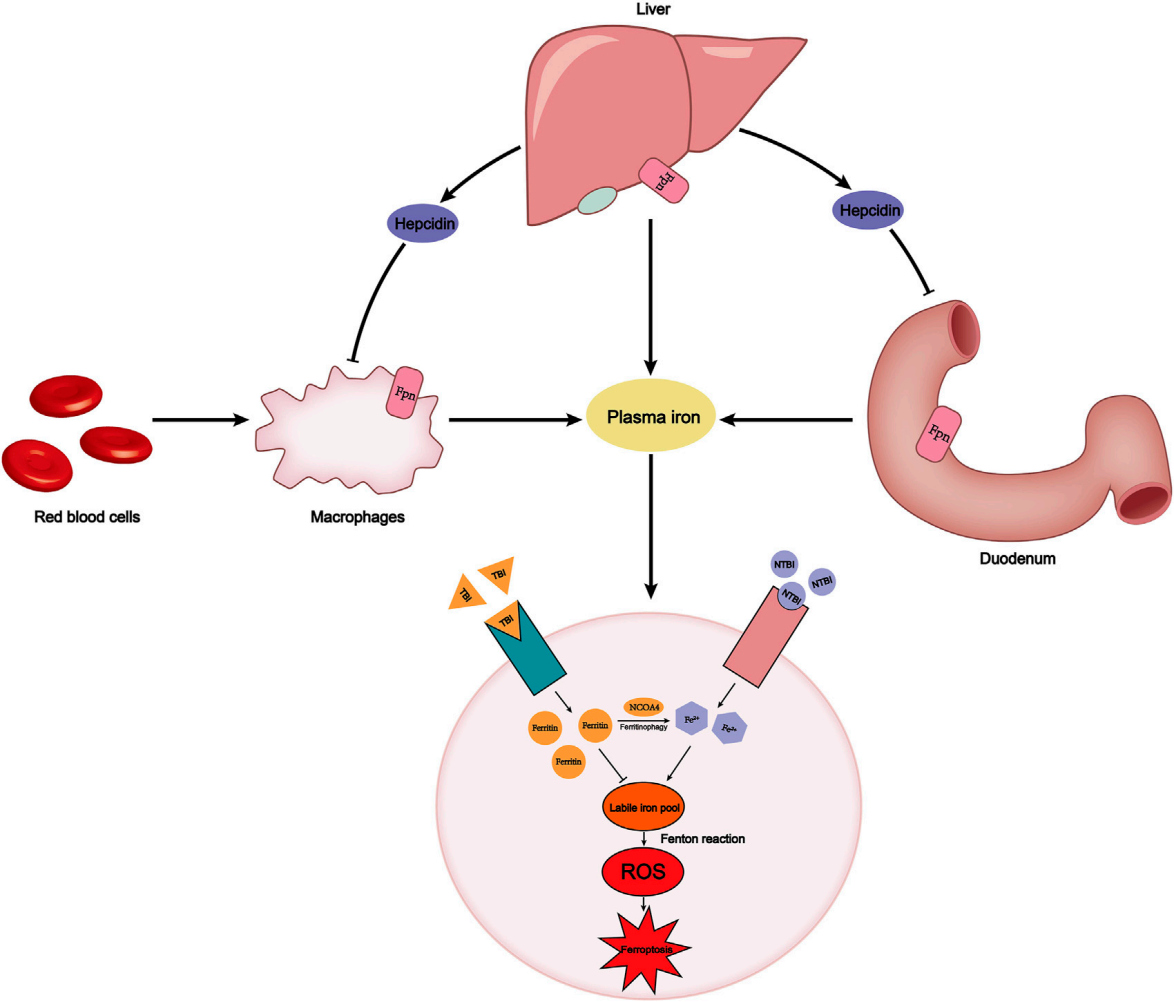
Plasma iron concentration is always maintained within a certain range. This relies on the precise dynamic balance of iron metabolism. FPN on the surface of macrophages, the duodenum, and liver transports iron into the plasma. The endogenous source of iron is mainly hemoglobin iron that is swallowed by macrophages, while the exogenous source is iron in food absorbed by the duodenum. Hepcidin secreted by the liver can degrade FPN to reduce iron export. Plasma iron is divided into TBI and NTBI according to whether it binds to transferrin. TBI binds to the TfR1 on the cell membrane surface and stores iron in the form of ferritin, avoiding the occurrence of Fenton reaction. However, labile iron pool, derived from NCOA4-mediated ferritinophagy or NTBI absorbed by SLC39A14, will cause an increase in intracellular ROS and ferroptosis. FPN, ferroportin; TBI, transferrin-bound iron; NTBI, non-transferrin-bound iron; TfR1, transferrin receptor.

Ferroportin (Fpn), also known as SLC40A1, is the only protein known to export nonheme iron to the outside of the cell. Fpn is widely distributed on the surface of duodenal epithelial cells, macrophages, and hepatocytes. The main source of iron in the human body is hemoglobin iron recovered by macrophages phagocytosing senescent red blood cells. The macrophages then transport the iron into plasma through Fpn. Simultaneously, the duodenum absorbs iron ions from food into the human body through Fpn (Figure 1) (Ganz, 2013). The lack of Fpn on the membrane surface of other tissues leads to intracellular iron enrichment (Donovan et al., 2005). Bao et al. found that Fpn was down-regulated, leading to accumulation of lipid peroxide and neuronal cell ferroptosis in a mouse model of Alzheimer’s disease (Table 1) (Bao et al., 2021). Hepcidin, a hepatic-secreted peptide hormone, binds to Fpn and promotes internalization and degradation of Fpn, resulting in the decreased export of non-heme iron (Nemeth et al., 2004). In addition to the Hepcidin-Fpn signaling pathway, the miR-124/Fpn signaling pathway has also been confirmed to affect the occurrence of ferroptosis. High levels of miR-124 in serum inhibit the expression of Fpn, leading to iron accumulation and ferroptosis in cells (Bao et al., 2020).

**TABLE 1:** The regulatory mechanism of ferroptosis.

Target	Regulation mechanism	Effect	References
Iron export	Hepcidin-Fpn pathway	Hepcidin promotes FPN internalization and degradation, causing iron accumulation in cells, which promotes ferroptosis	Bao et al. (2021)
	MiR-124/Fpn pathway	High serum levels of miR-124 inhibit FPN expression and promote ferroptosis	Bao et al. (2020)
Iron import	Serum transferrin level, NTBI and SLC39A14	When serum transferrin levels decrease, NTBI levels increase. NTBI accelerates cellular iron accumulation and ferroptosis. But at this time, SLC39A14, which is not the main transport protein under normal circumstances, can antagonize the effect of NTBI.	Yu et al. (2020)
	SREBF2 and transferrin	SREBF2 induces ferritin at the transcriptional level and inhibits ferroptosis	Hong et al. (2021)
	USP7/P53/TfR1 pathway	USP7 promotes P53 ubiquitination and increases TfR1 transcription. And TfR1 is one of the markers of ferroptosis	Tang et al. (2021b)
Iron storage and utilization	Ferritinophagy	NCOA4 facilitates degradation of ferritin in a selective cargo-mediated autophagy manner, which is an important source of iron for labile iron pool	Ito et al. (2021)
Other iron metabolism	HSF1/HSPB1/TfR1 pathway	HSF1 indirectly regulates cellular iron homeostasis and reduces the expression of TfR1 through HSPB1	Sun et al. (2015)
GPX4	Selenium metabolism	Selenium not only participates in the synthesis of GPX4 but also promotes the transcription of GPX4 through TFAP2C and SP1	Alim et al. (2019)
	mTORC1/4EBP/GPX4 pathway	MTORC1 and 4EBP act as a bridge between cystine, cysteine and GPX4, and can accelerate the synthesis of GPX4	Zhang et al. (2021)
System Xc^−^	ATF3 and ATF4 transcriptional regulation	ATF3 binds to the SLC7A11 promoter to inhibit transcription, while ATF4 does the opposite	Wang L et al. (2020), Chen et al. (2017)
	KEAP1/NRF2/SLC7A11	After separation of NRF2 from KEAP1, NRF2 enters the nucleus and binds with the ARE of the SLC7A11 promoter to up-regulate the expression of SLC7A11	Habib et al. (2015)
	BECN1 post-translational modification	BECN1 directly binds to SLC7A11 to inhibit its activity	Song et al. (2018)
	Amino acid metabolism	AMPK/SREBP1/BCAT2 pathway reduces intracellular glutamate concentration and inhibits system Xc^−^. Increasing the concentration of extracellular glutamate produce the same result	Gao et al. (2015); Wang et al. (2021)
	Immunotherapy and chemotherapy	IFNγ secreted by CD8^+^ cells inhibits SLC7A11 and SLC3A2. Radiotherapy can also interfere with the function of SLC7A11	Lang et al., (2019), Wang W et al. (2019)
FSP1-CoQ10-NADPH	MDM2 and MDMX	The MDM2-MDMX heterodimer can up-regulate the antioxidant system activity of FSP1-CoQ10	Venkatesh et al. (2020)
Lipid metabolism	ACSLs	ACSL4 promotes phosphatidylethanolamine containing AA and AdA to participate in phospholipid synthesis and increase the proportion of phospholipids that are prone to be oxidized. And ACSL1 adjusts the lipid composition by assembling αESA into DAG and TAG.	Doll et al. (2017) , Beatty et al. (2021)
	Lipoxygenase	ALOX12 catalyzes the oxidation of arachidonic acid. 15LOX and PEBP1 combine to form a complex and then oxidize PUFA to form 15-HpETE-PE, which is an important signaling molecule of ferroptosis	Chu et al., (2019), Anthonymuthu et al. (2021), Wenzel et al. (2017)
	MDM2 and MDMX	MDM2 and MDMX form a complex to inhibit the transcription factor PPARα, and up-regulate the sensitivity to ferroptosis	Venkatesh et al. (2020)

FPN, ferroportin; NTBI, non-transferrin-bound iron; USP7, ubiquitin-specific protease 7; TfR1, transferrin receptor 1; HSF1, heat shock factor 1; HSPB1, heat shock protein beta-1; GPX4, glutathione peroxidase 4; mTORC1, mechanistic target of rapamycin complex 1; ATF3, activating transcription factor 3; ATF4, activating transcription factor 4; KEAP1, Kelch-like ech-associated protein 1; NRF2, NF E2 Related Factor 2; ARE, antioxidant response element; AMPK, AMP-activated protein kinase; SREBP1, sterol response element binding protein 1; BCAT2, branched-chain amino acid aminotransferase 2; IFNγ, interferon γ; FSP1, Ferrop-Suppressor-Protein 1; ACSLs, acyl-CoA synthetase long-chain family; ACSL4, long-chain acyl-CoA synthetase 4; AA, arachidonic acid; AdA, adrenal acid; ACSL1, long-chain acyl-CoA synthetase 1; DAG, diacylglycerols; TAG, triacylglycerols; ALOX12, arachidonate 12-lipoxygenase; 15LOX, 15-lipoxygenase; PEBP1, phospholipid-ethanolamine binding protein-1; PUFA, polyunsaturated fatty acid; 15-HpETE-PE, 15-hydroperoxy-eicosatetraenoyl phosphatidylethanolamine.

### Iron Import

Under physiological conditions, ferric iron exported from cells binds to transferrin, which is a protein secreted primarily by the liver and transports iron to the bone marrow and other tissues. Conversely, if transferrin in plasma decreases, iron binds to albumin and anions to form non-transferrin-bound iron (NTBI), which can be absorbed by tissue cells, causing iron overload (Ganz, 2013). Yu et al. reported that when transferrin levels in serum decreased, the vulnerability of liver fibrosis in both humans and mice increased significantly, which was due to the accumulation of NTBI in liver cells and leads to ferroptosis of liver cells. In this case, SLC39A14, which is not the main transporter under physiological conditions, can act as a transporter for NTBI to induce ferroptosis (Yu et al., 2020). However, research has found that SREBF2, the principal regulator of cholesterol synthesis, can directly induce transferrin expression at the transcription level, reduce intracellular iron pools, and suppress ferroptosis induced by iron accumulation (Hong et al., 2021). Transferrin receptor 1 (TfR1) can transfer transferrin into cells through receptor-mediated endocytosis, and release iron carried by transferrin into the iron pool. TfR1 has been identified as a specific target antigen related to ferroptosis (Feng et al., 2020). The research by Tang et al. (2021a) demonstrated that Ubiquitin-specific protease 7 (USP7) promotes ubiquitination of P53, leading to increased transcription of TfR1 and ferroptosis (Tang et al., 2021a).

### Iron Storage and Utilization

Ferritin is an intracellular protein that oxidizes ferrous iron to ferric iron to avoid the occurrence of Fenton reaction, composed of light subunits (Ftl) and heavy subunits (Fth1). Ferritin is an inhibitor of ferroptosis, knockdown of Fth1 expression significantly increases the occurrence of ferroptosis (Rui et al., 2021). Ferritin is a form of iron storage in cells, and ferroptosis requires ferritin degradation to release stored iron. Quantitative proteomics found that NCOA4 can deliver ferritin to lysosomes and degrade ferritin in a selective cargo-mediated autophagy manner. Therefore, this process is called ferritinophagy (Mancias et al., 2014). Iron-independent ROS in the cell induces ferritinophagy and will increase intracellular iron levels (Park and Chung, 2019). The ferrous ions produced by ferritinophagy and the NTBI participate in the formation of labile iron pool in the cell, which is the chief culprit for the generation of ROS through the Fenton reaction (Ito et al., 2021). Therefore, iron storage and iron utilization are involved in the occurrence of ferroptosis.

### Other Iron Metabolism

Novel regulators of iron homeostasis have been described. Sun et al. found that phosphorylated heat shock protein beta-1(HSPB1) acted as a negative regulator of ferroptosis by reducing iron uptake by cells and the production of lipids. Transphosphorylation of HSPB1 increases ferritin expression and reduces TfR1 expression. HSPB1 is expressed in a heat shock factor 1(HSF1) dependent manner. Protein kinase C (PKC) regulates the phosphorylation of HSPB1 and indirectly regulates ferroptosis (Sun et al., 2015). The HSF1-HSPB1 pathway and PKC are novel regulators of iron homeostasis.

## Antioxidant Metabolism

### Glutathione Peroxidase 4

**FIGURE 2:**
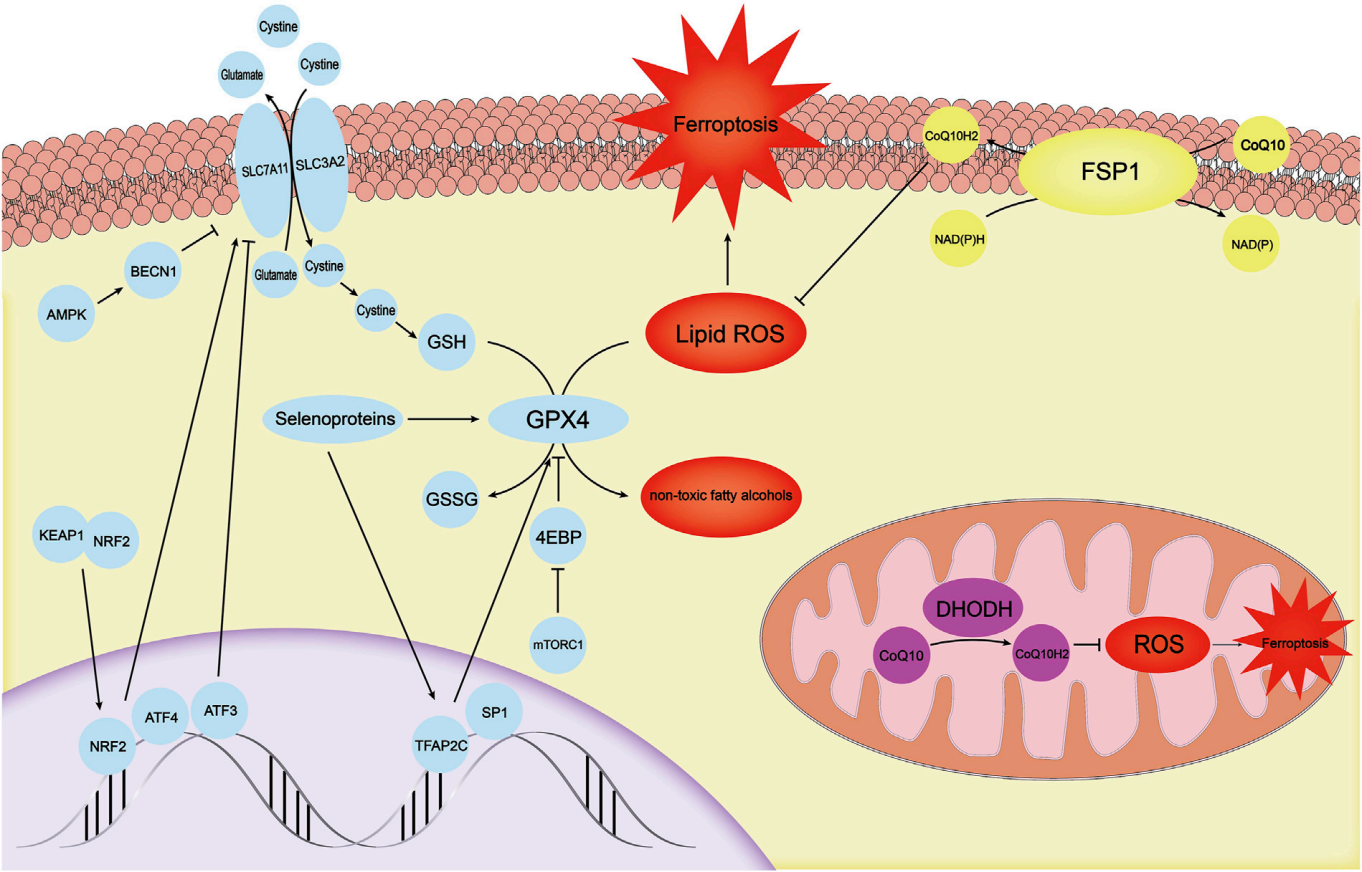
There are three independent antioxidant systems in cells. The antioxidant system with GPX4 as the core is the first antioxidant system discovered. It is regulated by mechanisms such as TFAP2C, SP1, mTOR-4EBP, and selenoproteins. SLC7A11, which provides a substrate for GPX4, is regulated by ATF3, ATF4, KEAP1-NRF2, and AMPK-BECN1. The FSP1-CoQ10-NADPH system, another antioxidant system in the cell membrane, can inhibit ferroptosis through CoQ10H2. DHODH primarily inhibits ROS in mitochondria by synthesizing CoQ10H2 to avoid the ferroptosis process in mitochondria. GPX4, glutathione peroxidase 4; mTOR, mechanistic target of the rapamycin complex 1; ATF3, activating transcription factor 3; ATF4, activating transcription factor 4; KEAP1, kelch-like ech-associated protein 1; NRF2, NF E2 Related Factor 2; AMPK, AMP-activated protein kinase; FSP1, Ferrop-Suppressor-Protein 1; NADPH, nicotinamide adenine dinucleotide phosphate; DHODH, dihydroorotate dehydrogenase; ROS, reactive oxygen species.

Not only does abnormal iron homeostasis regulation play an important role in ferroptosis but ferroptosis also requires the dysregulation of the intracellular antioxidant system. Yang et al. found that in ferroptosis cells, reduced glutathione (GSH), one of the most important endogenous antioxidants, was depleted, leading to the inactivation of glutathione peroxidase 4 (GPX4) (Yang et al., 2014). GSH-dependent GPX4 transforms toxic lipid peroxides in cells into non-toxic fatty alcohols, and prevents membrane phospholipids from reacting with ROS to produce lipid peroxides that directly lead to ferroptosis (Figure 2) (Magtanong et al., 2016). GSH is an essential substrate for GPX4 and exerts antiferroptosis activity. It is synthesized from cysteine and glutamic acid through a two-step enzymatic reaction and is oxidized to oxidized glutathione (GSSG) after reacting with lipid peroxides. Therefore, the antioxidant system comprising the GPX4 core is considered one of the most powerful regulators for protecting cells from ferroptosis. The appearance of a variety of diseases has been closely associated with the knockout of GPX4, such as neuronal degeneration (Hambright et al., 2017) and acute renal failure (Friedmann Angeli et al., 2014). Conversely, inhibiting GPX4 in drug-tolerant persisted cancer cells increases their vulnerability to ferroptosis. This discovery suggests a new treatment strategy for tumors (Hangauer et al., 2017; Viswanathan et al., 2017).

Selenoproteins are unique and rare proteins among a large protein family with unique physiological functions. Selenocysteine, which is not one of the 20 proteinogenic amino acids, is involved in the synthesis of GPX4. Selenocysteine ensures the need for unexpected antioxidant capacity (Ingold et al., 2018). Moreover, selenium is not only related to the synthesis of GPX4 but also promotes GPX4 transcription by coordinating and activating the transcription factors TFAP2C and SP1, allowing cells to respond to ferroptosis stimulation in an adaptive manner (Alim et al., 2019). In addition to the regulation of the transcription level, cystine and cysteine, as raw materials that simultaneously participate in the synthesis of GSH and GPX4, can activate the mechanistic target of rapamycin complex 1 (mTORC1) to inhibit the downstream 4EBP axis and promote GPX4 synthesis instead of indirectly regulating GSH levels (Zhang et al., 2021). In fact, mTORC1 acts as a bridge to link cystine and cysteine availability with the regulation of GPX4 and ferroptosis. Gaschler et al. recently discovered a new type of ferroptosis inducer FINO2 that inhibits GPX4. This is different from the class II ferroptosis inducer represented by RSL3. FINO2 can inhibit GPX4 while directly oxidizing polyunsaturated fatty acids (PUFAs), and provides a new perspective for the intervention of ferroptosis by adjusting GPX4 levels (Gaschler et al., 2018).

### System Xc^-^

System Xc^−^ is an amino acid reverse transporter located on the surface of the cell membrane, which can transport cystine outside the cell and glutamate inside the cell in a 1:1 ratio across the membrane. Therefore, System Xc^−^ activity is closely related to intracellular GSH content and GPX4 function. Ferroptosis is induced if system Xc^−^ activity is inhibited by erastin treatment (Dixon et al., 2012; Dixon et al., 2014). System Xc^−^ is a dimer composed of two subunits, SLC7A11 and SLC3A2, of which SLC7A11 makes a critical difference in limiting the rate of amino acid transport (Lewerenz et al., 2013). Multiple regulatory mechanisms at transcription, post-translational modification (PTMS), and protein levels determine the ability of System Xc^−^ to uptake cystine.

Activating transcription factors are a family of transcription factors that includes activating transcription factor 3 (ATF3) and activating transcription factor 4 (ATF4), which have been confirmed to be related to the function of the system Xc^−^ (Wang J et al., 2020; Chen et al., 2017). In the presence of the intracellular redox imbalance, the mRNA level of ATF3 increased. Activated ATF3 binds tightly to the SLC7A11 promoter, inhibits the expression of SLC7A11, and promotes the occurrence of ferroptosis in oxidatively stressed cells (Wang L et al., 2020). Interestingly, the effects of ATF4 is completely opposite. Increased expression of ATF4 up**-**regulates system Xc^−^ expression and increases cell resistance to ferroptosis (Chen et al., 2017). There are other transcription factors, such as the redox-sensitive transcription factor NF E2 Related Factor 2 (NRF2) and P53, which regulate the function of system Xc^−^ (Habib et al., 2015). In the basal state, Kelch-like ech-associated protein 1 (KEAP1) ubiquitinates NRF2, and when cells are under oxidative stress, NRF2 separates from KEAP1. Subsequently, NRF2 enters the nucleus and binds to the antioxidant response element (ARE) of the proximal promoter of its target gene SLC7A11 to up-regulate the expression of SLC7A11 (Habib et al., 2015). As the most famous tumor suppressor gene, P53 has unique and diverse functions in the regulation of ferroptosis. Under stimulation of low or moderate levels of ROS, P53 can prevent the accumulation of lethal ROS, but when high levels of ROS are produced, P53 causes ferroptosis in cells by inhibiting SLC7A11 transcription (Jiang et al., 2015). Multiple transcription factors can be considered indirect regulatory targets of ferroptosis.

Unlike regulation at the transcriptional level, BECN1 can regulate the activity of system Xc^−^ through post-translational modification. BECN1 binds directly to SLC7A11 to form the BECN1-SLC7A11 complex, rather than interfering with the transcription of SLC7A11. Prior to this, BECN1 requires phosphorylation at Ser90/93/96 *via* AMP-activated protein kinase (AMPK) (Song et al., 2018).

Amino acid metabolism is the main mechanism regulating protein levels, and both cysteine and glutamate within and outside the cell have an impact on system Xc^−^. High extracellular glutamate concentrations inhibit the activity of system Xc^−^, whereas elevated intracellular glutamate concentrations have the opposite effect. If cells are treated with erastin or sorafenib, the intracellular labile iron level will increase and subsequently induce ferritinophagy (Park and Chung, 2019). Wang et al. showed that ferritinophagy activated downstream AMPK and inhibited nuclear translocation of the sterol response element binding protein 1 (SREBP1), resulting in reduced transcription of the branched-chain amino acid aminotransferase 2 (BCAT2). BCAT2 is involved in the *de novo* synthesis of intracellular glutamate, and inhibition of BCAT2 reduces intracellular glutamate, which induces ferroptosis in cells (Wang et al., 2021). Similarly, inhibition of extracellular glutamine breakdown, which creates extracellular conditions of high glutamate, inhibits ferroptosis (Gao et al., 2015).

Recently, unique mechanisms by which immunotherapy and chemotherapy affect the system Xc^−^ have been reported. It was found that immune-activated CD8^+^T cells secreted interferon γ (IFNγ) that downregulated the expression of SLC7A11 and SLC3A2 expression (Lang et al., 2019; Wang W et al., 2019). SLC7A11 has also been shown to be a key target for the simultaneous action of immunotherapy and radiation therapy to induce ferroptosis (Lang et al., 2019). These novel studies provide a new research perspective to explore the mechanism of regulation of the system Xc^−^, and future studies may focus on the effect of the intervention of physiological factors.

### Other Parallel Antioxidant Mechanisms

Do cells have only one barrier against ferroptosis? Whether there is a parallel but completely independent antioxidant system with GPX4 and system Xc^−^ has become the key question regarding its antioxidant mechanism. Two research groups, Doll et al. (2019) and Bersuker et al. (2019) described independent mechanisms for cell resistance to ferroptosis almost simultaneously. The researchers determined that in some tumor cell lines, cells were able to resist ferroptosis even when the antiferroptosis system with the core of GPX4 was absent. Through gene sequencing of a large number of cells, the authors identified a previously unrecognized new mitochondrial-associated 2 apoptosis inducing factor (AIFM2), and renamed it “Ferrop-Suppressor-Protein 1” (FSP1). FSP1 must be recruited to the lipid membrane after myristoylation to perform its reductase function (Bersuker et al., 2019). The lipid membrane is rich in ubiquinone (CoQ10), and its reduced form, ubiquinol, can act as a lipophilic radical that traps antioxidant harvesting lipid peroxyl radicals (Doll et al., 2019). To reduce CoQ10, FSP1 must be supported by the coenzyme nicotinamide dinucleotide phosphate (NADPH), so FSP1-CoQ10-NADPH constitutes an independent antiferroptotic system.

In fact, cells contain other parallel antioxidant mechanisms. According to a recent study by Mao et al., there is a unique ferroptosis resistance system in mitochondria, which is dominated by dihydroorotate dehydrogenase (DHODH) (Mao et al., 2021). DHODH in GPX4-deficient cells leads to significant mitochondrial lipid peroxidation. Similarly, to the activity of the FSP1-CoQ10-NADPH axis, DHODH promotes the regeneration of CoQ10, which traps lipid peroxides in the mitochondrial membrane. Intervention with other parallel antioxidant mechanisms is a promising target to inhibit ferroptosis.

## Lipid Metabolism

The Fenton reaction triggered by abnormal iron metabolism leads to an increase in ROS, and an antioxidant system represented by GPX4 can eliminate ROS toxicity to cells, but the link between the two is lipid metabolism within the cell. Phospholipids are a component of cell membranes. ROS generated by the Fenton reaction tends to react with phospholipids (PL) to form hydroperoxy-phospholipids (PLOOH), which are the main elimination target of GPX4 (Magtanong et al., 2016). Phospholipids are lipids containing phosphoric acid, including a hydrophobic part composed of fatty acids and a hydrophilic part containing phosphoric acid. Phospholipids composed of polyunsaturated fatty acids are the main substrate for ROS oxidation (Kagan et al., 2017). Polyunsaturated fatty acids refer to straight chain fatty acids containing two or more double bonds, divided mainly into the omega-6 and omega-3 series (Wiktorowska-Owczarek et al., 2015). In the process of lipid synthesis, the first reaction using fatty acids is to catalyze the conversion of fatty acids into fatty acyl-CoA. The acyl-CoA synthetase long-chain family (ACSLs) is the first enzyme to catalyze the synthesis of phospholipids by acyl-CoA. Among them, long-chain acyl-CoA synthetase 4 (ACSL4) was identified as the key executor of ferroptosis (Yuan et al., 2016).

The preferred substrate for oxidation is phosphatidylethanolamine containing arachidonic acid (AA) and adrenal acid (AdA), and ACSL4 has a strong selectivity for AA-CoA and AdA-CoA (Doll et al., 2017; Dierge et al., 2021). ACSL4 promotes more peroxidable fatty acids to participate in the phospholipid synthesis process, and changes the composition ratio of fatty acids in lipids (Doll et al., 2017). Long-chain acyl-CoA synthetase 1 (ACSL1) adjusts the lipid composition by assembling αESA into diacylglycerols (DAG) and triacylglycerols (TAG). ACSL4 and ACSL1 adjust the content of non-conjugated fatty acids and conjugated fatty acids in lipids to adjust the vulnerability to ferroptosis. Unlike ACSL, lipoxygenase consists of a series of enzymes that catalyze the oxidation of PUFA double bonds to form lipid peroxides. Arachidonate 12-lipoxygenase (ALOX12) mainly catalyzes the oxidation of arachidonic acid, which is closely related to P53-mediated ferroptosis (Chu et al., 2019). P53 can indirectly activate ALOX12 by inhibiting SLC7A11 transcription, causing ferroptosis. It should be noted that although the transcription of SLC7A11 is reduced, it does not have a significant effect on GPX4 and GSH, indicating that this is an independent ferroptosis mechanism of GPX4 and ACSL4 (Chu et al., 2019), which involves 15-lipoxygenase (15LOX) and phospholipid-ethanolamine binding protein-1 (PEBP1) to combine and form a complex. The 15LOX/PEBP1 complex oxidizes various PUFAs to form 15-hydroperoxy-eicosatetraenoyl phosphatidylethanolamine (15-HpETE-PE), an important signaling molecule of ferroptosis (Wenzel et al., 2017; Anthonymuthu et al., 2021).

MDM2 and MDMX are negative regulatory proteins of the P53 gene. However, their role in ferroptosis has nothing to do with P53, but with lipid metabolism (Venkatesh et al., 2020). During ferroptosis, the MDM2-MDMX heterodimer formed by MDM2 and MDMX inhibits the activity of the transcription factor PPARα in a post-translational manner. The PPAR transcription factor family is a large-scale regulator of lipid components, and reduced PPARα activity increases susceptibility to ferroptosis. At the same time, the MDM2-MDMX heterodimer can up-regulate the antioxidant system activity of FSP1-CoQ10 (Venkatesh et al., 2020). In conclusion, the lipid composition of the cell membrane is both the substrate and site of ferroptosis execution, and the regulation of the lipid composition of the cell membrane is an independent mechanism able to regulate ferroptosis.

## Ferroptosis and Coronary Atherosclerotic Heart Disease

**FIGURE 3:**
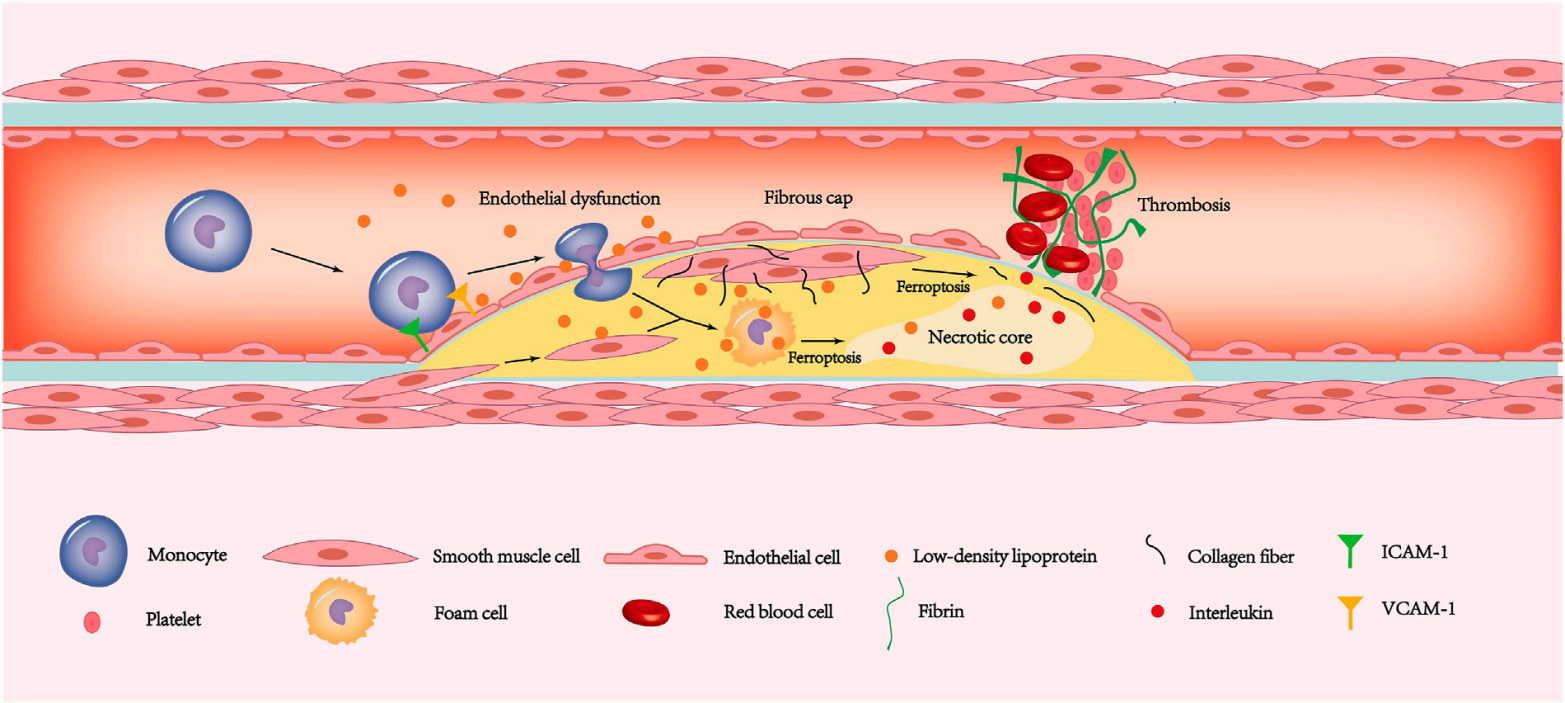
The ferroptosis of endothelial cells, smooth muscle cells and macrophages is involved in the pathogenesis of coronary atherosclerotic heart disease. The increased expression of ICAM-1 and VCAM-1 allows monocytes to adhere to the endothelial surface. Due to ferroptosis leading to endothelial dysfunction, monocytes have the opportunity to deform and enter the inner membrane. The monocytes under the inner membrane gradually transform into macrophages. Then some macrophages and smooth muscle cells phagocytose LDL and then form foam cells. In addition, some smooth muscle cells migrate to the plaque surface and secrete a large amount of extracellular matrix such as collagen fibers, forming thick fibrous caps. As the disease progresses, ferroptosis in smooth muscle cells leads to gradual thinning of the fibrous cap. Similarly, foam cells ferroptosis promotes the release of interleukins in the necrotic core. Exfoliation of the endothelium caused by ferroptosis exposes fibrinogen to the tissue. The combination of thin fibrous caps, endothelial stripping, and large amounts of interleukin released from the necrotic core leads to a rapidly forming thrombosis that blocks the coronary artery and causes ACS. LDL, low-density lipoprotein; ACS, acute coronary syndrome.

Coronary artery disease refers to a series of diseases with angina pectoris as the main symptom caused by the inability of the coronary blood supply to meet the metabolic needs of the myocardium. Coronary artery disease is a general term for a series of diseases that include coronary atherosclerotic heart disease, coronary artery spasm, and myocardial bridge—of which coronary atherosclerotic heart disease accounts for the vast majority. Coronary atherosclerotic heart disease is a disease characterized by hyperplasia of atherosclerotic plaques. In the early stage of atherosclerosis, endothelial cell ability to resist blood cell attachment is reduced under stimulation of high levels of low-density lipoprotein (LDL) (Verhoye and Langlois, 2009; Trpkovic et al., 2015), hypertension (Quyyumi and Patel, 2010) and inflammation (Weissberg and Bennett, 1999). The expression of adhesion molecules on the surface of endothelial cells is dramatically increased, and the permeability of endothelial cells and the subendothelial extracellular matrix are simultaneously altered (Figure 3). This process is called endothelial dysfunction. Circulating monocytes adhere to the surface of the artery intima under the impact of endothelial cell adhesion molecules and gradually penetrate the arterial intima (Libby et al., 2011). Monocytes entering the intima differentiate into mononuclear macrophages under stimulation of inflammatory factors. Under stimulation of inflammation, the smooth muscle cells of the arterial media are recruited into the intima and merge with smooth muscle cells that settle in the inner membrane (Libby et al., 2011).

Endothelial dysfunction also causes the lipid composition of the blood to gradually accumulate under the inner membrane. To remove deposited lipids, especially LDL, monocytes, macrophages, and smooth muscle cells will phagocytose lipids to form foam cells. As lipids and foam cells accumulate and lipid deposition under the inner membrane, a fatty streak gradually forms in the intima, which are the early lesions of atherosclerosis (Maguire et al., 2019; Wang D et al., 2019). However, the phagocytic ability of foam cells is limited and the deposited lipid cannot be engulfed completely. Therefore, a large number of cell components are wrapped by the lipid pool formed by extracellular lipids, and these components together become the basis for the formation of the lipid core. Long-term hypoxia and inflammation in the lipid core cause foam cells to die in a variety of ways, including necrosis and apoptosis. This process releases components such as cell debris, which constitute the main component of the necrotic core (Tabas, 2010). Smooth muscle cells secrete a large amount of extracellular matrix, including collagen fibers and elastin, covering the surface of the necrotic core, which is called the fibrous cap. The necrotic core and fibrous cap together constitute the pathological basis of coronary atherosclerotic heart disease and coronary atherosclerotic plaque formation.

As plaque size increases, coronary lumen stenosis reduces blood supply to cardiomyocytes and presents as angina pectoris. A potentially more lethal process may occur if the plaque thrombus suddenly occludes the coronary arteries and blood flow is drastically reduced. The produces a lack of collateral circulation compensation and can cause acute coronary syndromes (ACS), including unstable angina (UA) and acute myocardial infarction (AMI) (Crea and Libby, 2017). According to current opinions, the causes of acute thrombosis on the plaque surface are divided mainly into two types: plaque rupture (Davies and Thomas, 1984) and plaque erosion (Dai et al., 2018). Plaque rupture is the main cause of ACS. About two-thirds of ACSs are caused by plaque rupture (Arbustini et al., 1999). Plaques that are prone to rupture are called vulnerable plaques. Pathological features of vulnerable plaques include thin fibrous caps, large necrotic cores, and few smooth muscle cells (Finn et al., 2010). Macrophages cause vulnerable plaque rupture and thrombosis through a variety of regulatory cell death methods, including apoptosis (Hutter et al., 2004) and necroptosis (Karunakaran et al., 2016). Unlike macrophages, smooth muscle cells secrete an extracellular matrix to protect vulnerable plaques. The reduction in smooth muscle cells caused by multiple cell death pathways can significantly increase plaque vulnerability (Bennett et al., 2016).

Another vital pathogen that causes the occurrence of ACS is plaque erosion, which is responsible for up to about a third of ACS incidence. Compared to plaque rupture, plaque erosion has smaller necrotic cores, a higher number of smooth muscle cells, and thicker fibrous caps (Crea and Libby, 2017). In fact, the current explanation for the mechanism of plaque erosion is the two-hit hypothesis. De-endothelization of the plaque surface is the initial step of plaque erosion, which is the first hit. Endothelial cell dysfunction or death leads to the activation of platelets in the blood and the release of neutrophil extracellular traps (NETs) by neutrophils (Döring et al., 2017). The above factors are called the second hit, which induces thrombosis on the surface of the plaque. Through the study of the atherosclerosis process, it is easy to see that the death of various cells plays a key role in the occurrence and development of atherosclerosis. Current studies have confirmed that RCD methods such as apoptosis (Hutter et al., 2004), necroptosis (Karunakaran et al., 2016), and pyrolysis (Zhou et al., 2021) all occur in the plaque evolution process. Thus, do macrophages, smooth muscle cells and endothelial cells undergo ferroptosis during plaque progression?

As early as 30 years ago, Sullivan proposed the iron hypothesis, which pointed out that iron accumulation in atherosclerosis increases the risk of cardiovascular disease (Sullivan, 1981). The iron hypothesis is still controversial because the mechanism of iron accumulation leading to coronary atherosclerotic heart disease is not yet fully understood. The iron hypothesis has been widely questioned because hereditary hemochromatosis, a disease characterized by iron deposition throughout the body, not only reduces the risk of coronary atherosclerotic heart disease but also reduces the incidence (Mascitelli and Goldstein, 2014). Recent studies have shown that HFE gene mutations in patients with hemochromatosis regulate the expression of LDL receptors in liver cells and Kupffer cells. Under iron stimulation, HFE-deficient Kupffer cells phagocytose LDL-C in large amounts and reduce blood lipid levels (Demetz et al., 2020). Therefore, the reduced risk of coronary atherosclerosis in patients with hemochromatosis patients is actually related to the secondary low plasma level of LDL-C, which once again perfects and verifies the correctness of the iron hypothesis. Since the iron hypothesis was proposed, it has been widely recognized after continuous improvement, but the specific mechanism of iron deposition leading to atherosclerosis has not yet been fully elucidated.

The discovery of ferroptosis may explain the biological effects of iron accumulation. A recent study found that the SIRT1 autophagy axis in foam cells can inhibit ferroptosis induced by excessive iron (Su et al., 2021). By adding the exogenous iron agent ferric ammonium citrate to THP-1 cells, Su et al. confirmed that excessive iron induced ferroptosis in foam cells. Excess iron inhibits the SIRT1-autophagy axis of foam cells and reduces GPX4, both of which together induce foam cell ferroptosis (Su et al., 2021). Ferroptosis foam cells will release large amounts of inflammatory mediators such as IL-1β and IL-18. IL-1β and IL-18 belong to the IL-1 family of cytokines, and both are processed by caspase-1 for their activation (Pfeiler et al., 2019). Furthermore, early studies have confirmed that IL-1β and IL-18 can not only promote the progression of atherosclerosis (Mallat et al., 2001; Kirii et al., 2003) but also cause late plaque instability (Mallat et al., 2001) and induce myocardial infarction (Seta et al., 2000). Therefore, macrophage ferroptosis and foam cells derived from macrophage ferroptosis are present throughout the process, from early plaque formation to late plaque instability.

Regulating macrophage ferroptosis and foam cells derived from macrophages has become a novel target for the treatment of coronary atherosclerotic heart disease. Iron is a necessary condition for macrophage ferroptosis, and the most important pathway for macrophage iron regulation is the hepcidin-FPN axis (Wunderer et al., 2020). Hepcidin degrades FPN on the surface of macrophages, the only iron export protein, thus increasing the iron content of macrophages (Donovan et al., 2005). If hepcidin expression is suppressed, macrophages can excrete excess iron and cholesterol, which reduces foam cell formation and the progression of atherosclerosis (Saeed et al., 2012). When the iron content is increased or under inflammatory conditions, bone morphogenetic protein (BMP) stimulates the expression of hepcidin. Similarly, inhibition of BMP can also significantly delay atherosclerosis and vascular calcification (Derwall et al., 2012). Although current studies have confirmed the two facts that macrophage ferroptosis promotes the progression of atherosclerosis and hepcidin interferes with the process of atherosclerosis by regulating iron content, the link between macrophage ferroptosis and hepcidin has not been confirmed. Whether hepcidin can affect the development of atherosclerosis by regulating macrophage ferroptosis has become a current research hotspot.

Smooth muscle cells are the cellular components of the fatty streak and the most important source of extracellular matrix secretion in plaques. Unlike macrophages, smooth muscle cell death interferes mainly with plaque vulnerability by affecting the components of the fibrous cap on the surface of the plaque (Bennett et al., 2016). Smooth muscle cells secrete collagen fibers, the main component of the fibrous cap. As smooth muscle cells die through various pathways, the thickness of the fibrous cap becomes thinner due to reduced secretion and degradation of collagen fibers (Allahverdian et al., 2018). Thin cap fibrous atheroma is usually responsible for acute cardiovascular events. Therefore, when smooth muscle cells die in the form of ferroptosis caused by various triggers, such as smoking and diabetes, it is likely to promote plaque rupture and lead to ACS. Sampilvanjil et al. extracted the components of the gas phase of cigarettes, which are the main components of cigarettes that induce cardiovascular disease, mainly including acrolein and methyl vinyl ketone (Sampilvanjil et al., 2020). Smooth muscle cells treated with components of the gas phase of cigarettes showed obvious ferroptosis characteristics, such as increased expression of PTGS2, increased active oxygen content of lipids, and depletion of GSH. Smooth muscle cells overexpressing GPX4 cannot escape ferroptosis induced by gas phase extracts. However, iron chelators and N-Acetyl-L-cysteine (an antioxidant that can replace GSH) can prevent ferroptosis of smooth muscle cells, demonstrating that cigarettes can induce ferroptosis by depleting GSH within smooth muscle cells (Sampilvanjil et al., 2020). Moreover, ferroptosis can also promote vascular calcification to cause coronary atherosclerotic heart disease (Durham et al., 2018).

Coronary artery calcification is an independent risk factor for coronary atherosclerotic heart disease and is closely related to plaque rupture (Nicoll and Henein, 2014). Smoking, diabetes, metabolic syndrome, chronic kidney disease, and oxidative stress are closely related to smooth muscle cell-induced vascular calcification (Durham et al., 2018). Oxidative stress has been identified as an initiating factor for smooth muscle cell-mediated phenotypic transformation and calcium salt secretion (Byon et al., 2008). Ma et al. used palmitic acid to stimulate smooth muscle cells to construct an *in vitro* model of oxidative stress, and found that smooth muscle cells produce ferroptosis and promote calcification deposition (Ma et al., 2021). In terms of mechanism, the extracellular matrix protein periostin (POSTN) negatively regulates the expression of SLC7A11 by inhibiting the expression of the smooth muscle cell P53 gene. After pretreatment with metformin, the ferroptosis of smooth muscle cells was significantly reduced, and the calcification deposition disappeared. In conclusion, metformin activates the antioxidant system of smooth muscle cells to resist ferroptosis and secondary calcification through the Nrf2 signaling pathway, which finally confirmed the close connection between ferroptosis and vascular calcification. Preventing coronary heart disease by intervening in smooth muscle cell ferroptosis will become another promising independent therapeutic target.

It was reported that endothelial cell ferroptosis contributes to the activation of adhesion factor and thrombus formation. The mechanism that triggers endothelial dysfunction is mainly related to endothelial cell oxidative stress, and ferroptosis also plays a role in oxidized low-density lipoprotein (oxLDL)-induced endothelial cell dysfunction (Bai et al., 2020). When endothelial cell dysfunction occurs, adhesion molecules on the surface of endothelial cells are overexpressed, including the intercellular adhesion molecule-1 (ICAM-1) and the vascular cell adhesion molecule-1 (VCAM-1) (Habas and Shang, 2018). The ferroptosis inhibitor Fer-1 reversed ICAM-1 and VCAM-1 expression secondary to the reduction in ferroptosis of endothelial cells and restored vascular endothelial cell function. GPX4, as an important protein of cellular antioxidant system, plays an important protective role in ferroptosis. Surprisingly, the endothelial cell-specific knockout of GPX4 did not damage vascular homeostasis in mice with a normal diet (Wortmann et al., 2013). Similarly, *in vitro* treatment of vitamin E deficient medium resulted in GPX4 knockout endothelial cell ferroptosis. Under these conditions, when mice were fed a diet deficient in antioxidant vitamin E, thrombotic events occurred in almost all mice. This may indicate that we can avoid ferroptosis and endothelial cell dysfunction by regular intake of antioxidants such as vitamin E. Besides, when the human body inhales PM2.5, the molecular mechanisms of ferroptosis in endothelial cells as the first line of defense include GSH depletion combined with iron metabolism disorder (Wang and Tang, 2019). Zinc oxide nanoparticles (ZnONPs), widely used in cosmetics, rubber, and pigments, promote NCOA4-mediated ferritinophagy in endothelial cells (Qin et al., 2021). Ferroptosis of endothelial cells mediated by ferritinophagy causes endothelial dysfunction and intense vascular inflammation. In summary, endothelial cells ferroptosis plays an important role in promoting atherosclerosis, and inhibition of ferroptosis by improving antioxidant metabolism may be a new target for protecting atherosclerosis.

## Ferroptosis and Myocardial Ischemia Reperfusion Injury

Coronary artery subtotal occlusion or complete thrombosis-induced occlusion is likely to cause the onset of AMI. When AMI occurs, the blood supply to myocardial cells is interrupted and the cells are in a state of severe hypoxia. Unfortunately, the rapid improvement in blood supply in the ischemic myocardium can aggravate the degree of myocardial injury. Ischemia-reperfusion is one of the main mechanisms of myocardial injury (IRI) caused by AMI, and can be responsible for up to 50% of the infracted myocardial area (Lillo-Moya et al., 2021). The previous theory believed that IRI was related to xanthine oxidase and NADPH oxidase levels, and to mitochondrial activity, but new research shows that ferroptosis also plays an important role. (Tang et al., 2021b). found that only during the reperfusion period, the expression of ferroptosis markers MDA and ACSL4 was significantly increased, accompanied by a down-regulation of GPX4 expression in the Sprague-Dawley rats (SD) model of IRI (Tang et al., 2021a). SD rats injected with the ferroptosis inhibitor DFO before IRI have up-regulated GPX4 expression during reperfusion, accompanied by a decrease in myocardial infarction area and a decrease in creatine kinase (CK) levels. After ischemia-reperfusion, the up-regulation of USP7 expression promotes the progression of ferroptosis through the USP7/P53/TfR1 pathway (Tang et al., 2021b). TfR1 takes transferrin in the blood into cardiomyocytes and provides the initiating factor iron for cardiomyocyte ferroptosis. These studies confirmed that ferroptosis was involved in myocardial IRI. More importantly, ferroptosis of myocardial cells occurs primarily during reperfusion (Tang et al., 2021a).

Diabetes is one of the most important risk factors for AMI. The incidence of myocardial ischemia in patients with diabetes is significantly higher than in the normal population (Ndumele et al., 2016). If diabetic patients experience myocardial IRI, ferroptosis and stress of the endoplasmic reticulum can promote each other and aggravate myocardial IRI within a state of high glucose and hypoxia (Li et al., 2020). In the study, compared to the control group, the degree of myocardial damage in the diabetes group increased dramatically, accompanied by ferroptosis and endoplasmic reticulum stress. Application of the ferroptosis inhibitor Fer-1 or the endoplasmic reticulum stress inhibitor salubrinal or tunicamycin can significantly reduce ferroptosis and endoplasmic reticulum stress, demonstrating that ferroptosis and endoplasmic reticulum stress mutually induce and promote diabetic myocardial IRI.

Existing studies have confirmed the important role of ferroptosis in myocardial ischemia-reperfusion. Therefore, suppressing ferroptosis during ischemia-reperfusion may become a new therapeutic strategy to protect the myocardium. Before reperfusion, Feng et al. administered Lip-1 to mice, a potent specific inhibitor of ferroptosis, which reduced the level of VDAC1 and increased the activity of GPX4 (Feng et al., 2019). Lip-1 also has the ability to maintain mitochondrial function and reduce the area of myocardial infarction. A variety of VDACs are embedded on the surface of the outer mitochondrial membrane (OMM), including VDAC1, VDAC2, and VDAC3. Among them, the oligomerization of VDAC1 induced the occurrence of apoptosis. A mechanism by which erastin induces ferroptosis is that of binding directly to VDAC2 on the surface of the OMM, which changes the permeability of mitochondria (Keinan et al., 2013). Lip-1 induces the opposite effect of erastin and cannot act on VDAC2. Lip-1 shuts down VDAC1 on the surface of OMM and weakens the ability of the VDAC1/GRP75/IP3R1 complex to transport Ca^2+^ to mitochondria, which inhibits cardiomyocyte apoptosis. Moreover, Lip-1 can strongly eliminate ROS and significantly reduces the ferroptosis of myocardial cells (Keinan et al., 2013). Therefore, Lip-1 may be a drug with great potential to protect cardiomyocytes from IRI.

## Ferroptosis and Heart Failure

Subsequent to insufficient blood supply and myocardial IRI caused by coronary atherosclerotic heart disease, a considerable number of patients exhibit a reduction in the number of myocardial cells and present severely damaged cardiac contractile function (Yancy et al., 2013). HF is a highly heterogeneous disease and is the common final result of various cardiovascular diseases, including coronary heart disease, cardiomyopathy, IRI, valvular disease, and hypertension, which develop to the advanced stage (Lakhal-Littleton et al., 2015). In addition to the fact that ferroptosis plays a driving role in various diseases causing HF there are also unique factors that induce ferroptosis to accelerate the progression of HF. In the development of HF, the imbalance of cardiac iron homeostasis can accelerate ferroptosis-mediated HF. Various iron metabolism abnormalities ultimately affect the content of labile iron pool in cells. If the FPN expressed by cardiomyocytes is specifically knocked out, the mice will show a significant increase in left ventricular diameter with a decrease in the ejection fraction (Fang et al., 2020). The non-specific knockout of the Hamp gene, which encodes hepcidin, in mice has been reported to lead to increased iron accumulation in the mouse heart, but did not significantly lead to the onset of cardiovascular disease (Zheng et al., 2021). This is because after hepcidin knockout, although the overall iron content in the heart increased significantly, iron was deposited mainly in noncardiomyocytes, which is the key difference between the two models. Similarly, knocking out Fth in the mouse heart specifically causes intracellular iron metabolism disorders, which can also lead to severe myocardial hypertrophy and myocardial damage (Chen et al., 2019). In addition, the study by Ito et al. found that the mice that specifically knocked out NCOA4 had significantly stronger cardiac function than the wild group after performing transverse aortic constriction (Ito et al., 2021). Their study confirmed that knocking out NCOA4 reduces the occurrence of ferritinophagy, which provides a beneficial help for inhibiting labile iron pool. Other studies confirmed that overexpression of SLC7A11 or exposure to of Fer-1 treatment in mice abolished pathological changes in the myocardium, which showed that myocardial iron metabolism disorder caused heart failure through ferroptosis of myocardial cells (Stevenson et al., 2019).

The noncoding RNA circSnx12 can up-regulate the downstream target miR224-5p, and can combine with the 3′UTR region of FTH1 to down-regulate its expression (Zheng et al., 2021). In addition to iron metabolism disorders, there are other mechanisms involved in the process of HF. The receptor 4 (TLR4)-NADPH oxidase 4 (NOX4) pathway is up-regulated in cardiomyocytes of mouse models of HF (Chen et al., 2019). TLR4 is the key to activating inflammation, and activation of inflammation promotes the appearance of cardiomyocyte autophagy and the up-regulation of NOX4. NOX4 is the downstream executor of cardiomyocyte ferroptosis. Another study found that NOX4 in heart samples from patients with advanced HF of ischemic cardiomyopathy promoted myocardial inflammation and myocardial fibrosis, which can be mutually verified with the above study (Stevenson et al., 2019). Myocardial fibrosis is the pathological basis for the heart remodeling process in HF. Mixed lineage kinase 3 (MLK3), a member of the MAP3K family, causes myocardial fibrosis by promoting ferroptosis in the late stage of congestive HF (J. Wang et al., 2020). It is worth mentioning that the plant antioxidant puerarin is an effective inhibitor of ferroptosis during heart failure. Puerarin can inhibit NOX4 activity and, at the same time, up-regulate the expression of GPX4 and FTH1, and thus, it can delay cardiomyocyte damage and deterioration of heart function (Liu et al., 2018). In the future, including ferroptosis as a therapeutic target may represent an effective breakthrough in the treatment of heart failure.

## Ferroptosis and Clinical Application

With the development of basic research related to ferroptosis, combining ferroptosis with clinical applications has gradually become the next important research direction. The clinical application of ferroptosis-related targets is currently in its infancy. The current clinical research results may enlighten us several potential intervention targets in clinical applications. Since it is difficult to collect ferroptosis indicators in clinical practice, most studies focus on iron metabolity-related indicators, such as serum iron, serum ferritin, transferrin and soluble transferrin receptors. These targets with translational medicine potential could be key to controlling the progression of cardiovascular disease in the future.

Since Sullivan’s hypothesis, people have focused on the relationship between serum iron and atherosclerotic diseases (Sullivan, 1981). The incidence of acute myocardial infarction was significantly higher in patients with elevated serum ferritin than in patients with reduced serum ferritin (Moradi et al., 2015). However, lower ferritin levels did not improve all-cause mortality from cardiovascular disease (Zacharski et al., 2007). There are many factors influencing the level of serum ferritin. Under pathological conditions such as inflammation and tumors, the level of serum ferritin will increase significantly. Therefore, we need more accurate predictors to link ferroptosis with coronary atherosclerotic heart disease. The soluble transferrin receptor (sTfR) is derived from the cell membrane surface receptor through hydrolysis, and it exists as a complex with transferrin in the blood. Although the concentration of soluble ferritin receptors is not directly related to the risk of coronary atherosclerotic heart disease, the concentration of sTfR increases with the number of affected coronary arteries (Braun et al., 2004). Hepcidin concentration also has limited predictive value for the risk of future myocardial infarction or cardiovascular death (Zeller et al., 2018). However, intrinsic Iron release, defined as low levels of hepcidin (<24 ng/ml) and high levels of sTfR (≥2 mg/L), has been associated with a significant reduction in cardiovascular mortality in patients with coronary atherosclerotic heart disease (Ruhe et al., 2018).

Elevated levels of ferritin and hepcidin were highly associated with a higher risk of new-onset heart failure in women but not in men (Klip et al., 2017). Alternatively, disorders in iron intake and absorption in patients with heart failure often result in iron deficiency (ID). Iron not only causes ferroptosis in the body, but also assists in oxygen transport and mitochondrial function. Hence, iron supplementation has become a widely accepted treatment for heart failure patients with ID. Intravenous administration of ferric carboxymaltose for 1 year significantly improves the results of 6-min walking trials in both anemic and non-anemic patients (Ponikowski et al., 2015). In patients with heart failure with ID, serum iron metabolism-related proteins could not correctly predict cardiac ferroptosis levels and heart failure mortality. All in all, it is difficult to use ferroptosis indicators as accurate predictors or therapeutic targets in clinical practice so far.

## Discussion

In recent years, research on the occurrence of ferroptosis and disease has gradually improved. More and more mechanisms related to the regulation of ferroptosis have been discovered, such as the FSP1-CoQ10-NADPH axis and DHODH antioxidant system. But there are still many unsolved mysteries. To date, the major research directions have focused on ferroptosis and tumors. By studying the regulatory pathways that promote tumor cell ferroptosis, we can provide new directions to prolong the survival of tumor patients. In addition, degenerative diseases of the nervous system, liver disease, and kidney disease are also hotspots in ferroptosis research. Due to the high incidence and mortality of cardiovascular diseases, these have gradually become the number one killer of human health. However, our understanding of the pathogenesis and treatment of cardiovascular disease is limited. After the discovery of apoptosis, researchers have confirmed its important role in cardiovascular disease. With the improved understanding of regulatory cell death, other modalities of regulatory cell death, such as necroptosis and pyrolysis, are believed to occur in cardiovascular diseases. Research on ferroptosis and cardiovascular disease is still in its infancy, and most existing studies have confirmed the existence of ferroptosis in cardiovascular disease. As we gradually deepen our understanding of the role of ferroptosis, this mechanism will become a potential breakthrough point in the treatment of cardiovascular disease.

Research on the mechanism of ferroptosis is a prerequisite for clinical treatment and application. The iron hypothesis has been a controversial issue for many years since it was put forward. The recent discovery that iron metabolism disorder is the first step in ferroptosis may be a strategy to quell the controversial issue of iron hypothesis. According to previous theories, the antioxidant system with GPX4 at the core is the only anti-ferroptosis mechanism. The FSP1-CoQ10-NADPH axis and the DHODH antioxidant system suggest that ferroptosis may be regulated by a variety of independent antioxidant mechanisms. The chemical structure of phospholipids is also a target for regulating ferroptosis. The structure of the phospholipid bilayer membrane of different ratios of fatty acid composition gives cells different degrees of vulnerability to ferroptosis. The discovery that conjugated and nonconjugated fatty acids have different ferroptosis characteristics has provided a novel insight for a strategies for intervention, that is, in the future, it is possible to alter the proportion of fatty acids by optimizing dietary intake by the population as a strategy to achieve the goals of prevention and treatment.

In terms of basic research, ferroptosis has been investigated in terms of coronary atherosclerotic heart disease progression to IRI and even HF. Existing research is limited to *in vitro* cell-based experiments and animal studies, and there is almost no verification of the pathological development process mentioned above in human samples. Further verification of the role of ferroptosis in human specimens will be the goal of the next stage of research. Arrhythmia-related diseases have so far been the blind spot in ferroptosis studies. In the future, exploring whether ferroptosis is related to arrhythmia may be part of the research prospects.

In clinical application, iron metabolism indicators can only predict the risk of AMI or initial heart failure in a limited manner. Since iron metabolism-related indicators are interfered by many other factors in the human body, more independent predictors of ferroptosis need to be discovered in the future.

In summary, current research has preliminarily shown that ferroptosis is closely related to a variety of cardiovascular diseases. Regulating ferroptosis will be one of the most promising emerging targets to improve the prognosis and the survival rate of cardiovascular diseases.
